# Announcement: Metal Ions in Biological Systems, EUROBIC II

**DOI:** 10.1155/MBD.1994.289

**Published:** 1994


					METAL IONS
IN
BIOLOGICAL SYSTEMS
UROBIC II
Florence -Italy
August 30 -September 3, 1994
INTERNATIONAL ORGANIZING
COMMITTEE:
I. Bertini (Italy) (Chairperson); J. Reedijk (The Netherlands);
E. Rizzarelli (Italy); G. Sykes (UK); R. Thorneley (UK) (Secretary);
C. Veeger (The Netherlands).
ORGANIZING COMMITTEE:
R. Cipollini (Honorary Chairperson); L. Banci (Chairperson);
S. Aime; M. Coletta; A. De Flora; A. Fontana; G. Natile; M. Nicolini;
B. Pispisa; M. Rossi; B. Salvato; A. Scozzafava; G. Smulevich;
M.S. Viezzoli (Secretary).
EUROBIC is the European Bioinorganic Chemistry Conference and is organized by
scientists from several European countries. This is the second edition of a biennial
series on Metal Ions in Biological Systems. The conference is organized with the goal
to overcome the scientific barriers among the European scientists and to develop and
reinforce European scientific interactions. On the occasion of the meeting, the
European Bioinorganic medal to a brilliant promising scientist in this branch of Science
will be awarded. The conference will discuss on the role of metal ions in biological
systems and will cover several aspects of Bioinorganic Chemistry:
Model complexes
Structure-function of metalloproteins and metalloenzymes
DNA-metal ions interactions
Inorganic pharmacology
Metal ions and environment
Theoretical aspects
Advancements in the techniques
Electron transfer
289
Contrast agents
Membrane metalloproteins
Genetic of metalloproteins
The Conference will take place in Florence at Palaffari during the period Tuesday,
August 30 to Saturday, September 3, 1994.
All persons interested in the subject matter of the meeting are cordially invited to
attend.
The program include plenary lectures and session lectures. Posters will form an
integral part of the meeting and two separate poster sessions are planned.
The cost of the registration fee (within the deadline) is 400,000 Italian Life. The
deadline for the submission of abstracts and for the registration is April 30, 1994.
For further information please contact:
Prof. Lucia Banci
Department of Chemistry- University of Florence
Via Gino Capponi, 7
50121 Florence Italy
Phone: 391 55 2757550- Fax: 39/55 2757555
290

				

